# The triheme cytochrome PpcF from *Geobacter metallireducens* exhibits distinct redox properties

**DOI:** 10.1002/2211-5463.12505

**Published:** 2018-11-08

**Authors:** Marisa R. Ferreira, Joana M. Dantas, Carlos A. Salgueiro

**Affiliations:** ^1^ UCIBIO‐Requimte Departamento de Química Faculdade de Ciências e Tecnologia Universidade NOVA de Lisboa Caparica Portugal

**Keywords:** electron transfer, *Geobacter metallireducens*, multiheme *c*‐type cytochrome PpcF, NMR

## Abstract

Electrogenic bacteria, such as *Geobacter*, can couple the oxidation of carbon sources to the reduction of extracellular electron acceptors; such acceptors include toxic and radioactive metals, as well as electrode surfaces, making *Geobacter* a suitable candidate for applied use in bioremediation and bioenergy generation. *Geobacter metallireducens* is more promising in this regard than the better studied *Geobacter sulfurreducens*, as it has more efficient Fe (III) reduction rates and can convert nitrate to ammonia. The operon responsible for nitrate reductase activity in *G. metallireducens* includes the gene encoding the cytochrome PpcF, which was proposed to exchange electrons with nitrate reductase. In the present work, we perform a biochemical and a biophysical characterization of PpcF. Spectroscopic techniques, including circular dichroism (CD), UV‐visible, and nuclear magnetic resonance (NMR), revealed that the cytochrome is very stable (*T*_m_ > 85 °C), contains three low‐spin hemes, and is diamagnetic (*S* = 0) and paramagnetic (*S* = 1/2) in the reduced and oxidized states, respectively. The NMR chemical shifts of the heme substituents were assigned and used to determine the heme core architecture of PpcF. Compared to the PpcA‐family from *G. sulfurreducens*, the spatial disposition of the hemes is conserved, but the functional properties are clearly distinct. In fact, potentiometric titrations monitored by UV‐visible absorption reveal that the reduction potential values of PpcF are significantly less negative (−56 and −64 mV,* versus* the normal hydrogen electrode at pH 7.0 and 8.0, respectively). NMR redox titrations showed that the order of oxidation of the hemes is IV‐I‐III, a feature not observed for *G. sulfurreducens*. The different redox properties displayed by PpcF, including the small redox‐Bohr effect and low reduction potential value of heme IV, were structurally rationalized and attributed to the lower number of positively charged residues located in the vicinity of heme IV. Overall, the redox features of PpcF suggest that biotechnological applications of *G. metallireducens* may require less negative working functional redox windows than those using by *G. sulfurreducens*.

**Abbreviations**
CD: circular dichroism
EXSY: EXchange SpectroscopY
*G. metallireducens*: *Geobacter metallireducens*
*G. sulfurreducens*: *Geobacter sulfurreducens*
HMQC: Heteronuclear Multiple Quantum Coherence
MALDI‐TOF‐MS: matrix‐assisted laser desorption ionization time‐of‐flight mass spectrometry
NHE: normal hydrogen electrode
NMR: nuclear magnetic resonance
NOESY: Nuclear Overhauser Effect SpectroscopY
PpcF: *G. metallireducens* cytochrome encoded by gene *Gmet0335*
TOCSY: TOtal Correlation SpectroscopY

Electrogenic bacteria can couple the oxidation of carbon sources to the reduction of extracellular electron acceptors. These include, for example, toxic and radioactive metals, as well as electrode surfaces from which electricity can be harvested in microbial fuel cells 1. Therefore, electrogenic bacteria are targeted for practical application in bioremediation and bioenergy fields. Particularly, *Geobacter*‐based applications are being developed due to their natural environmental abundance and high density current production on microbial fuel cells 2, 3.

*Geobacter sulfurreducens* was the first Geobacteraceae family member for which a genetic system was developed 4. Consequently, much information on the electron transfer components in this bacterium has been collected, including the identification of key *c*‐type cytochromes in the extracellular electron transfer respiratory pathways and their functional and structural characterization 2, 5, 6. Compared to *G. sulfurreducens*, the bacterium *Geobacter metallireducens* presents additional interest since (a) it is capable of remediate environments contaminated with aromatic compounds 7, (b) it has more efficient Fe (III) reduction rates 8, (c) it is able to convert nitrate to ammonia, and (d) it has more conductive pili 9. In addition, *G. metallireducens* is also much more versatile regarding the carbon sources 10. The large variety of carbon sources that can be used by *G. metallireducens* opens additional routes to be explored for practical applications in bioremediation, bioenergy, and even in the direct interspecies electron transfer 11, 12, 13. Therefore, understanding the electron transfer mechanisms underlying the distinct processes carried out by *G. metallireducens* is important. The genome of this bacterium was sequenced 8, and a genetic system is now available 7. *G. metallireducens* genome encodes for 65 multiheme cytochromes, which are numerically comparable to the 73 multiheme cytochromes encoded by the genome of *G. sulfurreducens* 14. However, genetic and proteomic studies indicate that the essential *c*‐type cytochromes for cellular growth in the presence of insoluble and soluble electron acceptors are distinct in the two bacteria 7. This also extends to the well‐conserved PpcA‐family of *c*‐type triheme periplasmic cytochromes that are amongst the most abundant in *Geobacter* species 2, 15. These are small proteins (approximately 10 kDa molecular weight) and are designated PpcA‐E in *G. sulfurreducens* 16. Similarly, a family composed by five triheme periplasmic cytochromes was also identified in *G. metallireducens* 8. However, in this case, they were designated PpcA, PpcB, PpcC, PpcE, and PpcF. The PpcF designation results from its lowest amino acid sequence identity with cytochrome PpcD from *G. sulfurreducens* (Table 1). The predicted cellular localization of these cytochromes at the periplasm indicates that, as in *G. sulfurreducens*, the triheme cytochromes are the most likely reservoir of electrons toward outer membrane electron transfer components 5, 6. Amongst all *c*‐type cytochromes identified in *G. sulfurreducens*, the PpcA‐family cytochromes are the best characterized 5.

**Table 1:** Amino acid sequence identity (%) between the PpcA‐family from *Geobacter sulfurreducens* and *Geobacter metallireducens*. The percentage of identity was obtained from the basic local alignment search tool (BLAST) 38

	*G. sulfurreducens*				
	A	B	C	D	E
*G. metallireducens*					
A	80	73	64	68	62
B	68	72	57	68	63
C	59	56	79	42	51
E	54	61	52	55	69
F	62	58	57	55	57

As mentioned, and contrarily to *G. sulfurreducens*,* G. metallireducens* cells are able to respire nitrate 17, 18. The operon responsible for the nitrate reductase activity in *G. metallireducens* includes the gene encoding to cytochrome PpcF, which was proposed to exchange electrons with the nitrate reductase enzyme 8. The participation of PpcF in the nitrate respiratory pathway, which has no counterpart in *G. sulfurreducens*, might explain its lower amino acid sequence identity compared to *G. sulfurreducens* cytochromes (Table 1). In this work, we undertake a biochemical and a biophysical characterization of PpcF using complementary spectroscopic techniques, namely UV‐visible, circular dichroism (CD), and NMR.

## Materials and methods

### Amino acid sequence analysis and cloning of cytochrome *ppcF* gene

Genomic DNA from *G. metallireducens* GS‐15 was kindly provided by D. R. Lovley (University of Massachusetts, Amherst). The *ppcF* gene sequence (GenBank accession number ABB30578) was identified by searching the *G. metallireducens* genome database on Kyoto Encyclopedia of Genes and Genomes Web site (http://www.genome.jp/kegg/genome.html), under the accession number T00295. The gene sequence was analyzed using the SignalP server 19 to predict the signal peptide cleavage, which is located after residue Ala^22^.

DNA fragment coding for the mature sequence of cytochrome PpcF was amplified from the *G. metallireducens* GS‐15 genomic DNA with primers containing restriction sites for the enzymes NotI (5′‐CACATGCGGCCGCCGCCGACGTATTTGAATTC‐3′) and HindIII (5′‐GCCCCTACCGCAGGTTCAAGCTTTTACTTCTTGTGGCAG‐3′). Fragment size (305 bp) was confirmed by 0.8% agarose gel electrophoresis. The DNA fragment was digested with the restriction enzymes NotI and HindIII and cloned into vector pVA203 15, 20. The resulting plasmid was designated pCS0335. Colonies were screened by colony PCR with primers (5′‐GGCTCGTATGTTGTGTGGAA‐3′ and 5′‐AAGGGAAGAAAGCGAAAGGA‐3′) and then were grown in liquid LB medium supplemented with ampicillin (100 μg·mL^−1^) for plasmid extraction and sequencing (STABvida Caparica, Portugal). *Escherichia coli* strain DH5α was used for cloning, and *E. coli* strain BL21‐DE3, co‐transformed with plasmid pEC86, was used for protein production. Primers were purchased from Invitrogen, restriction enzymes, and T4 DNA ligase from Fermentas (Waltham, MA, USA). All PCR products were purified using Wizard PCR Preps DNA Purification System (Promega, Fitchburg, WI, USA). Digested vector and plasmids were purified using E‐gel Electrophoresis System (Invitrogen, Waltham, MA, USA) and NZYMiniprep kit (NZYTech, Lisbon, Portugal), respectively. Phusion High‐Fidelity DNA polymerase (Finnzymes, Waltham, MA, USA) was used for amplification from genomic DNA and Taq DNA polymerase (VWR, Radnor, Pennsylvania, USA) for colony PCR.

### Expression and purification

The expression and purification of cytochrome PpcF was carried out as previously described for the PpcA‐family cytochromes from *G. sulfurreducens* and PpcA from *G. metallireducens* 21, 22. Briefly, *E. coli* BL21‐DE3 cells, which have the plasmid pEC86 that encodes for cytochrome *c* maturation gene cluster and the chloramphenicol resistance gene, were co‐transformed with the plasmid pCS0335, encoding for the mature cytochrome and carrying an ampicillin resistance gene. The cells were grown at 30 °C in 2xYT media containing chloramphenicol (34 μg·mL^−1^) and ampicillin (100 μg·mL^−1^), up to an OD_600nm_ of 1.5. At this point, protein expression was induced with 10 μm isopropyl β‐d‐thiogalactoside. After an overnight incubation also at 30 °C, cells were harvested by centrifugation at 6400 ***g*** for 20 min at 4 °C and resuspended in 30 mL of lysis buffer (100 mm Tris/HCl (pH 8), 0.5 mm ethylenediaminetetraacetic acid, and 20% sucrose) per liter of cell culture, containing 0.5 mg·mL^−1^ lysozyme. The periplasmic fraction was recovered by centrifugation at 14 700 ***g*** for 20 min (4 °C). The obtained supernatant was then ultra‐centrifuged at 225 000 ***g*** for 1 h (4 °C). The obtained supernatant was dialyzed against 2 × 5 L of 10 mm Tris/HCl (pH 8) and loaded onto 2 × 5 mL Bio‐Scale Mini UNOsphere S cartridges (Bio‐Rad, Hercules, CA, USA), equilibrated with the same buffer. The protein was eluted with a sodium chloride gradient (0–300 mm), and the obtained fraction was concentrated to 1 mL and injected in a Superdex 75 molecular exclusion column (GE Healthcare, Chicago, IL, USA) equilibrated with 100 mm sodium phosphate buffer (pH 8). Protein purity was evaluated by Coomassie‐stained SDS/PAGE.

### Determination of extinction coefficient and protein quantification

The concentration of the purified cytochrome was determined with the Pierce BCA Protein Assay Kit (Thermo Scientific, Waltham, MA, USA) using PpcA from *G. sulfurreducens* as standard, as previously described 22. UV‐visible absorption spectra were acquired in the range 350–700 nm for the cytochrome, at room temperature, using a Thermo Scientific Evolution 201 spectrometer. Fully oxidized and reduced spectra were measured using quartz cuvettes with 1 cm path length (Hellma, Hellma analytics, Müllheim, Germany). The reduced samples were prepared by the addition of sodium dithionite. The molar extinction coefficient of cytochrome PpcF in the reduced form was determined at 553 nm (87 mm ^−1 ^cm^−1^).

### Determination of molecular mass and heme quantification

The theoretical molecular mass of cytochrome PpcF was calculated from the amino acid composition of the mature protein plus the molecular mass of three heme *c*‐type groups, using the pI/Mw tool program on the ExPASy Server (https://web.expasy.org/compute_pi/), as previously described 22. Briefly, the experimental molecular mass was determined by matrix‐assisted laser desorption ionization time‐of‐flight mass spectrometry (MALDI‐TOF‐MS) using a Voyager‐DE™ PRO Biospectrometry workstation equipped with a nitrogen laser radiating at 337 nm from Applied Biosystems (Waltham, MA, USA). A matrix solution of sinapinic acid in 70 : 30 water/acetonitrile with 0.1% TFA (final concentration) was used. The measurements were performed in triplicated in a positive ion mode using the cytochrome *c* (MW 12 361.96 Da) from Sigma‐Aldrich (St. Louis, MO, USA) as internal calibration.

The *c*‐type heme quantification in cytochrome PpcF was determined by the pyridine hemochrome method 23. An aqueous solution containing 5 μm of protein was incubated with 50 mm NaOH and 20% v/v pyridine at room temperature for 15 min. Then, the obtained solution was separated in two fractions. One fraction was reduced with sodium dithionite, whereas the other was oxidized with potassium ferricyanide yielding the pyridine ferrohemochrome and pyridine ferrihemochrome forms, respectively. UV‐visible absorption spectra were acquired for both forms, and the quantification of the number of *c*‐type hemes was obtained using the difference absorption coefficient of 21.84 mm ^−1 ^cm^−1^ at 550 nm for the pyridine ferrohemochrome and the pyridine ferrihemochrome 23.

### Circular dichroism spectroscopy

Circular dichroism experiments were performed on a JASCO J‐810 spectropolarimeter equipped with a Peltier‐thermostated cell support. CD spectra were acquired for 70 μm protein samples at pH 8.0. The temperature was controlled to ± 0.1 °C. CD spectra were the average of four scans obtained by collecting data at 0.5‐nm intervals and scan rate of 50 nm per minute from 200 to 260 nm. After recording a CD spectrum at 25 °C, the thermal unfolding with a linear temperature increase at 3 °C min^−1^ (from 25 to 95 °C) was followed by recording the ellipticity variation at 235 nm. The CD spectra were recorded at 95 °C and after cooling back the sample to 25 °C. All spectra and signals were corrected for the buffer contribution.

### Redox titrations and determination of the macroscopic reduction potential values

Redox titrations of cytochrome PpcF were followed by visible spectroscopy at 15 °C inside an anaerobic glove box (MBraun, Garching, Germany) with oxygen levels kept under 1 ppm, as previously described 22. Samples (30 μm final concentration) were prepared in 80 mm phosphate buffer, with NaCl (250 mm final ionic strength), at pH 7.0 and 8.0. The following mixture of redox mediators was added to the solution with a final concentration of approximately 1.5 μm, as described in the literature 24, to ensure the equilibrium between the redox center of the protein and the working electrode: methylene blue, gallocyanine, indigo trisulfonate, indigo tetrasulfonate, indigo disulfonate, anthraquinone‐2,6‐disulfonate, anthraquinone‐2‐sulfonate, 2‐hydroxy‐1,4‐naphthoquinone, safranine O, diquat, benzyl viologen, neutral red, and methyl viologen. These mediators cover the potential range of +21 to −440 mV [relative to the normal hydrogen electrode (NHE)].

To check for hysteresis, the redox titrations were performed in the reductive and in the oxidative direction at each pH. Sodium dithionite and potassium ferricyanide solutions were used to reduce and oxidize the samples, respectively. A combined Pt/Ag/AgCl electrode was used for measuring the solution potential values, which were then corrected to the NHE. The redox potential of the solution was measured after each addition of reductant or oxidant agent, and once stable, a visible spectrum was recorded using an Evolution 300 (Thermo Scientific) spectrophotometer. The experiments were performed at least twice, and the reduction potentials were found to be reproducible within ± 5 mV. The reduced fraction of the protein was determined by integrating the area of the α‐peak (553 nm) above the line connecting the flanking isosbestic points (545 and 559 nm) to subtract the optical contribution of the redox mediators, as described previously 25. Therefore, during the redox titration, any eventual shift in the absorption maxima of the α‐peak is taken into account for the determination of the reduced fraction values.

Each measured solution redox potential value was corrected to the NHE reference by the addition of 207 mV.

### NMR spectroscopy

All NMR spectra were acquired in a Bruker Avance III 600 spectrometer equipped with a triple‐resonance cryoprobe (TCI) at 15 or 25 °C and processed using TOPSPIN (BrukerBiospin, Karlsruhe, Germany). The ^1^H chemical shifts were calibrated using the water signal as internal reference and the ^13^C chemical shifts were calibrated through indirect referencing 26.

Cytochrome PpcF samples (with approximately 0.5 mm final concentration) were prepared in 80 mm phosphate buffer (pH 7.0 or 8.0) with NaCl (250 mm final ionic strength) in ^2^H_2_O. The reduced samples were obtained by adding gaseous hydrogen in the presence of catalytic amounts of Fe‐hydrogenase isolated from *Desulfovibrio vulgaris* (Hildenborough), as previously described 25. Partially oxidized samples used for NMR redox titrations were obtained by first removing the hydrogen from the reduced sample with nitrogen and then by adding controlled amounts of air into the NMR tube with a Hamilton syringe through the rubber cap.

To assist the heme signal resonance assignments, 2D ^1^H, ^1^H NOESY (Nuclear Overhauser Effect SpectroscopY) and TOCSY (TOtal Correlation SpectroscopY) NMR spectra were acquired for fully reduced and oxidized samples. In the fully reduced state, 2D ^1^H, ^1^H NOESY (80‐ms mixing time) and TOCSY (60‐ms mixing time) were acquired over a spectral width of 12 kHz. In the fully oxidized form, the same type of spectra were acquired with 80‐ and 45‐ms mixing time (spectral width of 41 kHz), respectively. In addition, 2D ^1^H,^13^C HMQC (Heteronuclear Multiple Quantum Coherence) NMR spectra were acquired for the oxidized sample (41 and 250 kHz in ^1^H and ^13^C dimensions, respectively). All 2D NMR spectra were analyzed with Sparky (TD Goddard and DG Kneller, Sparky 3, University of California, San Francisco, USA).

Samples of cytochrome PpcF, with approximately 70 μm final concentration, were used to monitor the individual heme oxidation patterns. This was achieved by acquiring a series of 2D ^1^H, ^1^H EXSY (EXchange SpectroscopY) NMR spectra at pH 7.0 (25‐ms mixing time), with the sample poised at different degrees of oxidation.

## Results and Discussion

The cytochrome PpcF from *G. metallireducens* is composed by 70 amino acids and has an isoelectric point (pI) of 8.96, as determined by the pI/Mw tool program on the ExPASy Server (http://web.exp-asy.org/compute_pi/). The high pI value of PpcF facilitates its purification by a cationic exchange chromatography. In fact, nearly pure fractions were obtained, at approximately 47% of the 300 mm NaCl gradient applied, before being injected onto the gel filtration column for final polishing. After this step, the protein purity was evaluated by SDS/PAGE electrophoresis and mass spectrometry. The protein yield was 1 mg per liter of cell culture medium. The mass spectrum obtained for the pure cytochrome showed an intense and well‐resolved peak correspondent to a molecular mass of 9737 Da (data not shown). This value is in excellent agreement with the expected value (9736, resulting from 7886 Da calculated for the cytochrome amino acids plus 1851 Da correspondent to the molecular weight of three heme groups). The presence of three heme groups was further confirmed by the pyridine hemochrome assay.

### Spectroscopic features and stability

**Figure 1:**
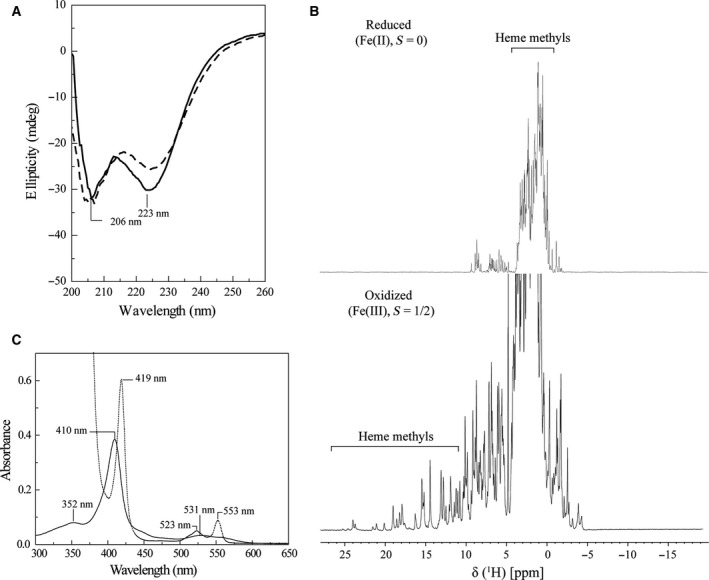
Spectroscopic properties of cytochrome PpcF from *Geobacter metallireducens* (pH 8.0). (A) Far UV‐CD spectrum in the native state at 25 °C (solid line) and upon heating to 95 °C (dashed line). The more intense negative bands, typical of α‐helix, are labeled; (B) 1D ^1^H NMR spectra of the reduced (upper) and oxidized (lower) forms (25 °C); (C) UV‐visible spectra of the fully oxidized (solid line) and reduced (dashed line) forms (25 °C).

CD and NMR spectroscopy were used to evaluate the correct fold of cytochrome PpcF. The latter technique, together with UV‐visible spectroscopy, was also used to identify the heme spin‐state features of the protein. The far UV‐CD spectra obtained at 25 and 95 °C are indicated in Fig. 1A. At 25 °C, the CD spectrum displays the typical features of a folded protein with high α‐helix content, as depicted by the intense negative bands at 206 and 223 nm. The slightly displacement of these two negative bands compared to the typical values observed for α‐helix at 208 and 222 nm is probably due to the considerable contribution of the three heme groups in such a small protein. The spectrum also presents a local maximum at 213 nm and a positive ellipticity above 250 nm. Upon heating to 95 °C, small linear changes in ellipticity were observed at 235 nm, except for the signal at 223 nm, which gets about 20% weaker, and for the local maximum at 213 nm, which redshifts slightly to 216 nm.

**Figure 2:**
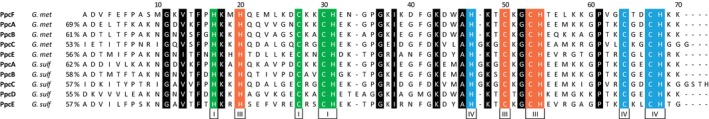
Alignment of the amino acid sequences of the periplasmic triheme cytochromes from *Geobacter sulfurreducens* and *Geobacter metallireducens*. The conserved residues in the proteins are boxed and colored (green, orange, and blue for hemes I, III, and IV, respectively) and black, for the heme‐attached and non‐heme‐attached, respectively. The heme numbering and the respective attached residues are indicated at the bottom of the last cytochrome amino acid sequence. The percentage of sequence identity relative to PpcF from *G. metallireducens* obtained from the basic local alignment search tool (BLAST) 38 is also indicated.

Upon re‐cooling to 25 °C, the final CD spectrum is essentially equivalent to the initial spectrum, indicating that the protein is very stable. The comparison between the amino acid sequences of the periplasmic triheme cytochromes from *G. metallireducens* and *G. sulfurreducens* suggests that the heme groups in cytochrome PpcF are covalently linked to the polypeptide chain and have bis‐histidinyl axial coordination (Fig. 2). Therefore, it is expected that the protein shows high stability (*T*_m_ > 85 °C), as it was also observed for all PpcA‐family cytochromes from *G. sulfurreducens* 15.

1D ^1^H NMR spectra of PpcF showed well‐dispersed and narrow signals, which further confirms the correct fold of the protein (Fig. 1B). The spectra obtained show a typical pattern of low‐spin heme groups 27. In fact, the NMR signals cover the spectral region 25 to −5 ppm and 11 to −3 ppm, in the oxidized and reduced form, respectively. Therefore, the protein is diamagnetic when reduced (Fe^2+^, *S* = 0) and paramagnetic when oxidized (Fe^3+^, *S* = ½). The spin state of the hemes was further confirmed by UV‐visible spectroscopy (Fig. 1C). The optical absorption spectrum of the oxidized cytochrome has maxima at 352, 410, and 531 nm. Upon reduction, the protein shows the Soret, β, and α peaks at 419, 523, and 553 nm, respectively. This spectral pattern is typical of low‐spin hexacoordinated hemes 22, 28.

### Specific assignment of the heme proton NMR signals in the reduced state

**Figure 3:**
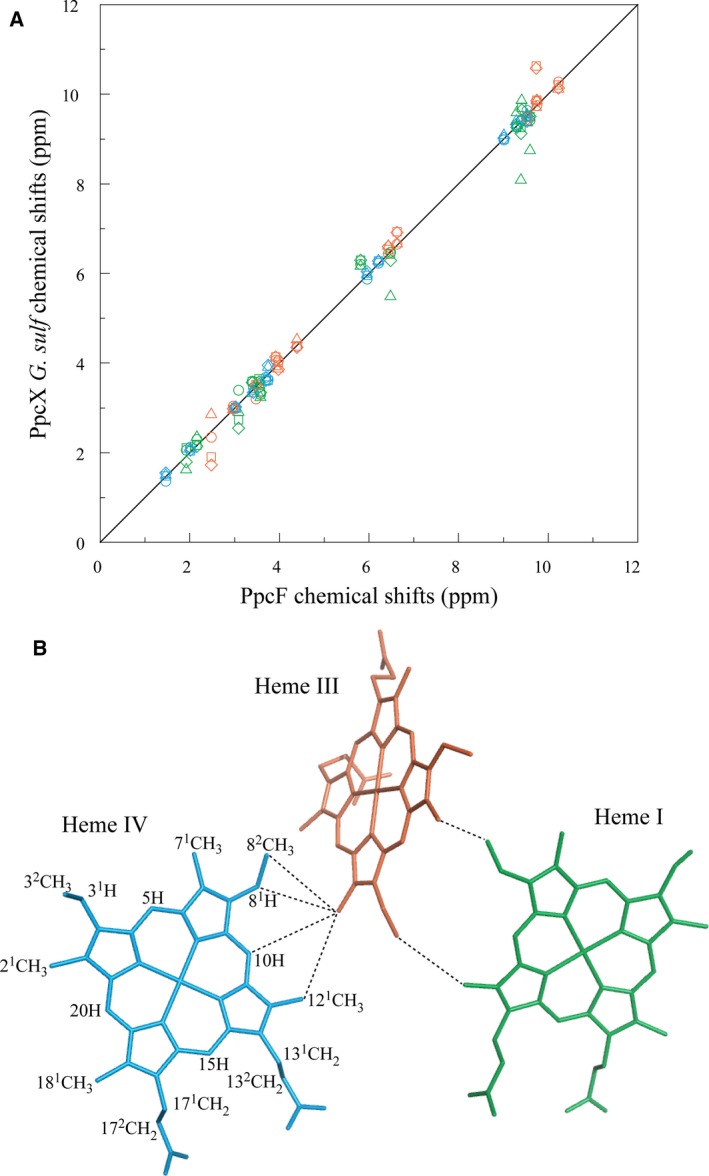
Analysis of the heme core architecture of cytochrome PpcF from *Geobacter metallireducens*. (A) Comparison between the observed heme proton chemical shifts of PpcF and those of cytochromes PpcX (X = A, B, D, or E) from *Geobacter sulfurreducens* 25, 30. Diamonds, squares, triangles, and circles correspond to cytochromes PpcA, PpcB, PpcD, and PpcE, respectively. Green, orange, and blue symbols correspond to hemes I, III, and IV, respectively. The solid line has a unit slope. The root mean square deviations (rmsd) between the heme proton chemical shifts measured for PpcF and those of PpcA, PpcB, PpcD, and PpcE are 0.26, 0.24, 0.35, and 0.13, respectively. (B) Interheme NOE connectivities (dashed lines) observed between the heme proton signals of PpcF from *G. metallireducens*. The heme core represented corresponds to that of PpcE from *G. sulfurreducens* (PDB code: http://www.rcsb.org/pdb/search/structidSearch.do?structureId=3H34 15), which showed the smaller rmsd values compared to PpcF. The IUPAC nomenclature for tetrapyrroles 39 is illustrated on heme IV.

The heme proton NMR signals of cytochrome PpcF in the reduced form were assigned using the strategy previously described for low‐spin multiheme cytochromes 29. The small size of the protein, with a ratio of approximately 20 residues per heme group, makes these signals excellent probes to monitor structural variations in the architecture of the heme core since they are essentially dominated by the intrinsic and extrinsic heme ring‐current effects. Consequently, the heme chemical shifts of cytochrome PpcF (Table S1) was compared to those previously determined for the *G. sulfurreducens* PpcA‐family members 25, 30 (Fig. 3A). The good correlation observed indicates that the overall heme core architecture is conserved. The slightly larger deviations observed for the heme proton signals of PpcD are not surprising since the structure of PpcD differs the most from those of the other family members 15. The maintenance of the heme core architecture in PpcF was further confirmed by the analysis of the interheme NOE connectivities in the 2D ^1^H,^1^H NOESY NMR spectrum (see dashed lines in Fig. 3B).

### Determination of the order of oxidation of the heme groups

**Figure 4:**
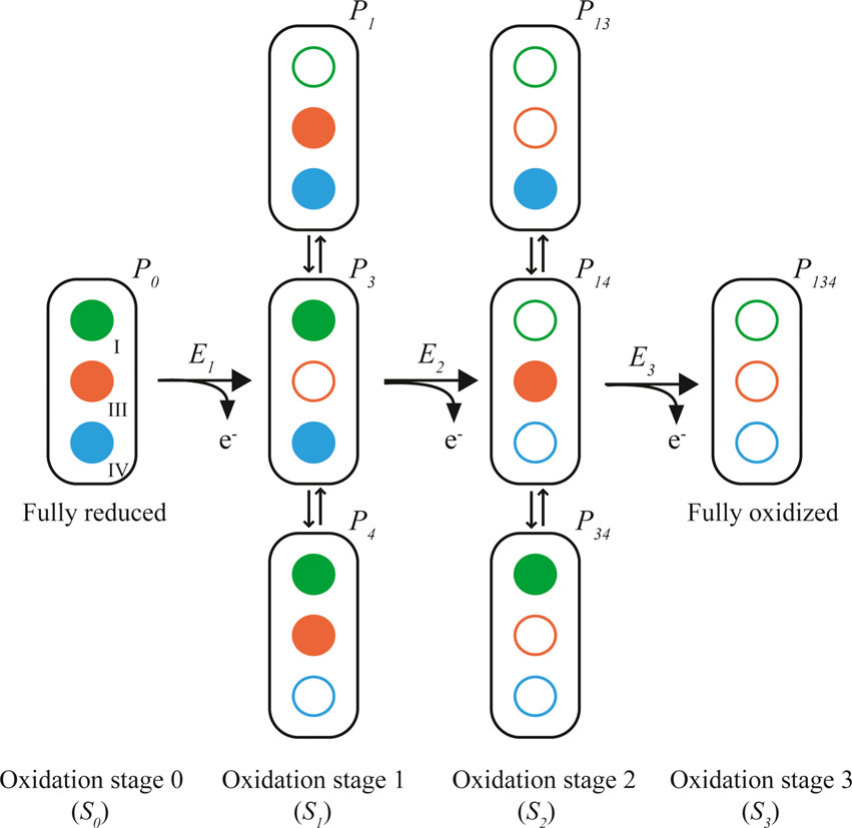
Schematic representation of the electronic distribution for a triheme cytochrome. The circles in each microstate represent the heme groups (green, orange, and blue for hemes I, III, and IV, respectively), which can be either reduced (filled circles) or oxidized (open circles). The microstates are grouped, according to the number of oxidized hemes, in four oxidation stages connected by three one‐electron redox steps (for a review, see 34). *P*_0_ represents the reduced microstate. *P*_*ijk*_ correspond to the microstates with heme(s) *i*,* j*, and *k* oxidized. *E*_1_, *E*_2_, and *E*_3_ are the macroscopic reduction potentials for the first, second, and third oxidation steps, respectively.

**Figure 5:**
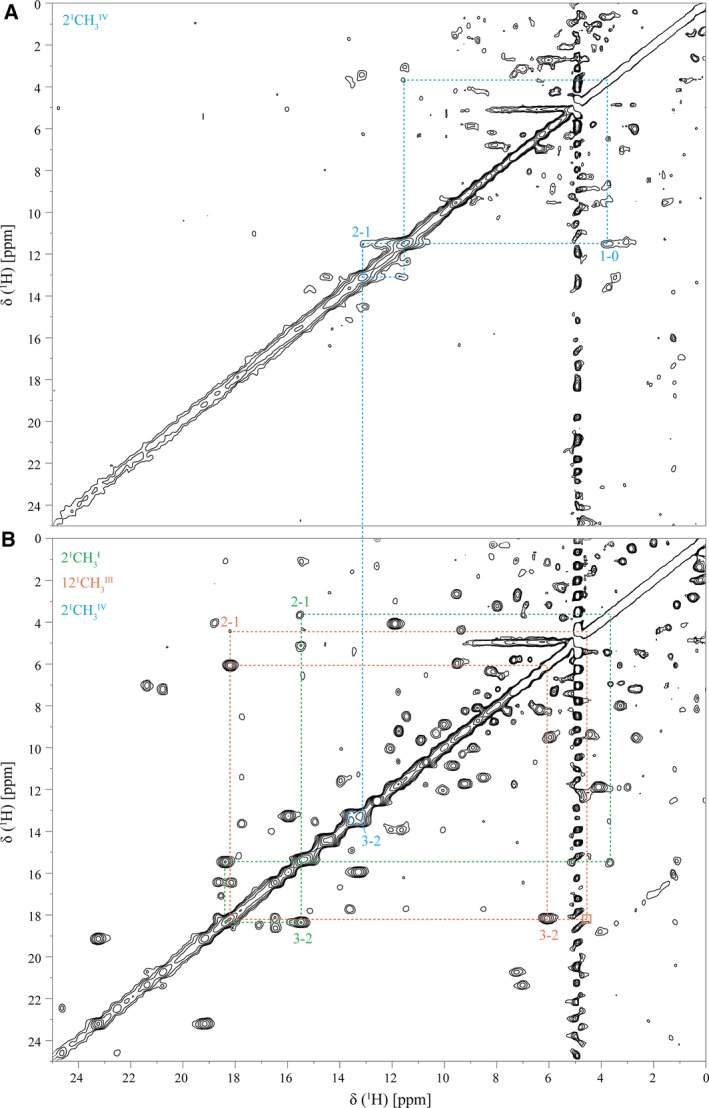
2D ^1^H,^1^H EXSY NMR spectrum of cytochrome PpcF obtained at intermediated oxidation levels (15 °C and pH 7.0). (A) Early stages of oxidation. (B) Later stages of oxidation. Cross‐peaks connecting the chemical shifts of the heme methyls in different oxidation stages are indicated by dotted lines. The Roman and Arabic numbers indicate the heme group and the oxidation stage, respectively. The empty box corresponds to the position of the weak connectivity of 12^1^ CH_3_ ^III^ connecting the oxidation stages 1–3 in *F*_2_ dimension.

Previous studies have shown that despite the highly conserved heme core architecture in different multiheme cytochromes families, as it is the case of tetraheme cytochromes *c*_3_ and triheme cytochromes *c*_7_, the redox behavior of the heme groups may be quite distinct 30, 31. Triheme cytochromes display three consecutive reversible steps of one‐electron transfer that convert the fully reduced to the fully oxidized state (Fig. 4). Consequently, four different redox stages are defined, each containing the microstates with the same number of oxidized hemes. In the particular case of fast intramolecular electron exchange (between the different microstates within the same oxidation stage) and slow intermolecular electron exchange (between different oxidation stages), relative to the NMR timescale, the individual heme substituent signals can be discriminated in the different oxidation stages (for a review see 30). Thus, the individual redox patterns of the hemes can be monitored by 2D ^1^H,^1^H EXSY NMR experiments. From all heme substituents, the NMR signals of the heme methyls are the easiest identifiable, making them ideal candidates to monitor the stepwise oxidation of the hemes 32. In fact, as depicted in Fig. 1B, the chemical shift of the heme signals is considerably different in the reduced and oxidized states, shifting from crowded regions in the fully reduced to relatively empty regions in the fully oxidized spectra. Therefore, in the present work, 2D ^1^H,^1^H EXSY NMR experiments were used to determine the order of oxidation of the heme groups in the cytochrome PpcF (Fig. 5). The unambiguous assignment of the 12 heme methyl signals in the fully reduced protein (Table S1) constitutes an excellent starting point to monitor their chemical shift variation up to their final position in the fully oxidized state. In the 2D ^1^H,^1^H EXSY NMR spectrum acquired in samples placed at early stages of oxidation, only signals connecting the chemical shift of heme IV methyls in oxidation stages 0 and 1 could be observed. This is illustrated for heme methyl 2^1^CH_3_^IV^ in Fig. 5A. The 2D ^1^H,^1^H EXSY NMR spectrum acquired for a more oxidized sample allowed us to follow the chemical shift of this methyl to its final position (Fig. 5B) and, consequently, to determine the profile of oxidation of heme IV (Table 2). The data obtained showed that the first step of oxidation is essentially dominated by the oxidation of heme IV (79%), which prevented the observation of well‐resolved connectivities for the other two heme groups (Fig. 5A). In fact, the very small percentage of oxidation of these hemes in the first redox step (7 and 11% for hemes III and I, respectively), results in a small variation in their chemical shifts between oxidation stages 0 and 1, so that the connectivities are located at or near the diagonal of the NMR spectra. Therefore, it was not possible to monitor the stepwise oxidation for hemes I and III starting from the reduced stage. In order to attain information relative to the stepwise oxidation of these two hemes, the assignment of all heme methyl signals was independently obtained in the fully oxidized state following the strategy previously described 33. The assignment of the heme methyl signals in the fully oxidized state (see Fig. S1 and Table S2) provided unambiguous starting points to monitor the stepwise oxidation of hemes I and III. In fact, in the 2D ^1^H,^1^H EXSY NMR spectrum acquired for samples where oxidation stages 1, 2, and 3 co‐exist, connectivities between the heme methyl signals were observable. This is illustrated for heme methyls 2^1^CH_3_^I^ and 12^1^CH_3_^III^ in Fig. 5B.

**Table 2:** Redox dependence of the heme methyl chemical shifts and heme oxidation fractions in cytochrome PpcF (pH 7.0 and 15 °C). The heme methyls 2^1^CH_3_^I^, 12^1^CH_3_^III^, and 2^1^CH_3_^IV^ were chosen to monitor the heme oxidation profiles. The selected methyls point outside the heme core (Fig. 3B) and, therefore, the contribution from the oxidation of neighboring hemes to their chemical shifts is minimized. The heme fractions of oxidation, *x*_*i*_, in each oxidation stage (*S*_0_ *–S*_3_; each containing the microstates with the same number of oxidized hemes, as defined in Fig. 4) were calculated accordingly to the equation *x*_*i*_ = (δ_*i*_ − δ_0_)/(δ_3_ − δ_0_), where δ_*i*_, δ_0_, and δ_3_ are the chemical shift of the heme methyls in stages *i*, 0, and 3, respectively

Oxidation stage	Chemical shift (ppm)			*x*_*i*_			Σ *x*_*i*_
	I	III	IV	I	III	IV	
*S*_0_	3.42	3.51	3.73	0	0	0	0
*S*_1_	5.14	4.47	11.55	0.11	0.07	0.79	0.97
*S*_2_	15.49	6.09	13.10	0.81	0.18	0.95	1.94
*S*_3_	18.38	18.20	13.59	1	1	1	3

**Figure 6:**
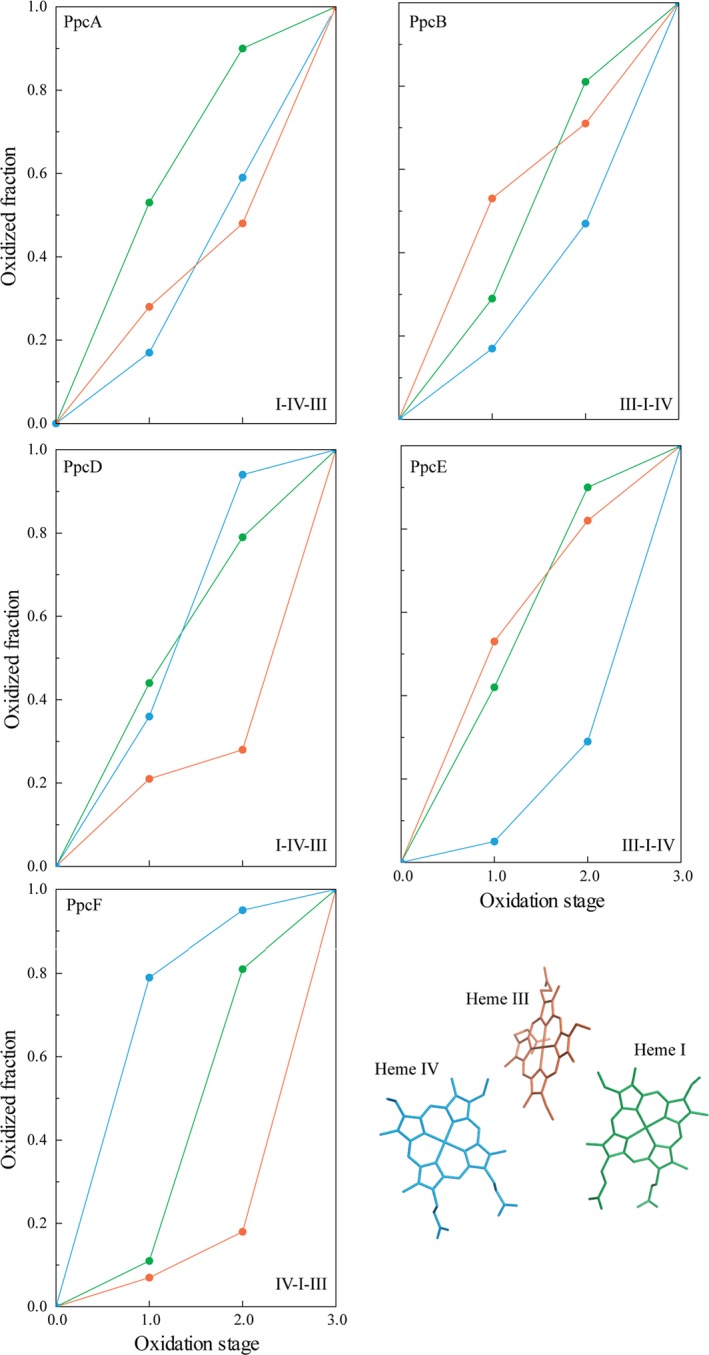
Heme oxidation fraction values of the cytochrome PpcF from *Geobacter metallireducens* and PpcA‐family cytochromes from *Geobacter sulfurreducens* (15 °C and pH 7.0). Data for hemes I, III, and IV are colored green, orange, and blue, respectively. The heme oxidation fractions for the *G. sulfurreducens* cytochromes are indicated in the Table S3. The heme core of PpcE from *G. sulfurreducens* is indicated (PDB code: http://www.rcsb.org/pdb/search/structidSearch.do?structureId=3H34 15).

The chemical shifts of heme methyls 2^1^CH_3_^I^, 12^1^CH_3_^III^, and 2^1^CH_3_^IV^ were used to calculate the oxidation fraction of each heme at different stages of oxidation (Table 2). The analysis of this table shows that heme IV becomes more oxidized in the first redox step (79%), followed by heme I (70%) in the second and by heme III in the last one (82%). Therefore, the order of oxidation of the heme groups in cytochrome PpcF is IV‐I‐III. Despite the high homology between PpcF and the triheme cytochromes from *G. sulfurreducens*, the heme oxidation profiles are quite distinct (Fig. 6). In fact, heme IV is never the first heme to oxidize in triheme cytochromes from *G. sulfurreducens*, and also, the percentage of oxidation of the dominant heme in each oxidation step is considerable smaller compared to the ones observed for PpcF (Fig. 6).

### Redox titrations followed by UV‐visible spectroscopy

**Figure 7:**
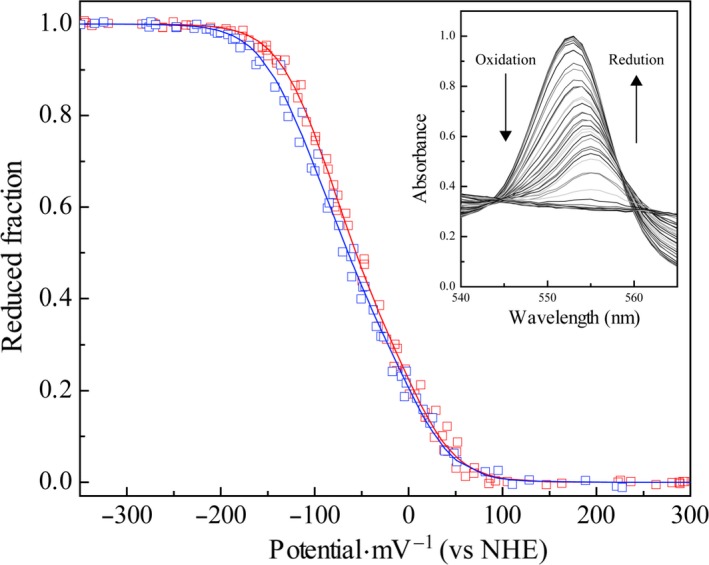
Redox titration curves for cytochrome PpcF from *Geobacter metallireducens* at different pH values (15 °C). The red and blue symbols refer to pH 7.0 and 8.0, respectively. The lines indicate the results of the fits for the three macroscopic reduction potentials (*E*_1_, *E*_2_, and *E*_3_), which are indicated in Table 3. The inset represents an expansion of the α‐band region at pH 8.0.

The significant differences observed between the heme oxidation profiles of cytochrome PpcF and *G. sulfurreducens* cytochromes prompt us to determine for PpcF the redox potential values in the physiological pH range (Fig. 7). The superimposition of the reductive and oxidative curves obtained at pH 7.0 and 8.0 indicates that the redox process is fully reversible, since no hysteresis is observed. The apparent midpoint reduction potential (*E*_app_) and the macroscopic reduction potentials for the first (*E*_1_), second (*E*_2_), and third (*E*_3_) oxidation steps (see Fig. 4) are listed in Table 3. Strikingly, despite the high level of amino acid sequence homology between PpcF and *G. sulfurreducens* cytochromes, as well as their conserved heme core, the values of PpcF are considerable less negative. Compared to pH 7.0, the values are smaller at pH 8.0, a behavior that was also observed for the cytochromes from *G. sulfurreducens*. The observed pH dependence is expected in electrostatic terms, since deprotonation of residues in the vicinity of the heme groups destabilizes the reduced state. The difference in the *E*_app_ values, obtained from the visible redox titrations carried out at pH 7.0 and 8.0 for PpcF (Table 3), also indicates that the redox‐Bohr effect is considerable smaller compared to PpcA and PpcD from *G. sulfurreducens*, suggesting that PpcF is unlikely to participate in electron/proton coupling mechanisms in *G. metallireducens*. In fact, the important redox‐Bohr effect observed for PpcA and PpcD from *G. sulfurreducens* indicated that they are functionally designed to couple e^‐^/H^+^ transfer at the physiological pH range for cellular growth 34. Moreover, the detailed structural analysis carried out for PpcA showed that the origin of the redox‐Bohr effect is explained by a network of hydrogen bonds established between the heme IV propionates groups (P_13_ and P_17_) and two positively charged lysines (Lys^7^ and Lys^9^) 35. Strikingly, Lys^7^ and Lys^9^ are conserved in *G. sulfurreducens* cytochromes, but are absent in cytochrome PpcF being replaced by a serine and glycine, respectively (see Fig. 2). Moreover, site‐directed mutagenesis studies have shown that the redox‐Bohr effect displayed by the members of the PpcA‐family in *G. sulfurreducens* seems to be modulated by the nature of the residue in position 6 36. In fact, cytochromes that have a conserved leucine in this position (PpcA and PpcD) showed higher redox‐Bohr effect compared to cytochromes that have a phenylalanine in an equivalent position (PpcB and PpcE) 36. Interestingly, PpcF also has a phenylalanine residue in position 6 (cf. Fig. 2 and Table 3). Overall, the absence of the positively charged residues Lys^7^ and Lys^9^, as well as the presence of the aromatic residue in position 6, might explain the smaller redox‐Bohr effect observed in PpcF.

**Table 3:** Macroscopic (*E*_1–3_) and apparent midpoint (*E*_app_) reduction potentials of cytochrome PpcF from *Geobacter metallireducens* (*Gm*) and PpcA‐family cytochromes from *Geobacter sulfurreducens* (*Gs*). The *E*_app_ values correspond to the point at which the oxidized and reduced fractions are equal. *E*_1_, *E*_2_, and *E*_3_ are the macroscopic reduction potentials for the first, second, and third oxidation steps, as described in Fig. 4

Cytochrome	*E*_app_ (mV)		*E*_1_ (mV)		*E*_2_ (mV)		*E*_3_ (mV)	
	pH		pH		pH		pH	
	7.0	8.0	7.0	8.0	7.0	8.0	7.0	8.0
PpcF *Gm* (this work)	−56	−64	−108	−125	−59	−66	10	6
PpcA *Gs* 25	−117	−138	−171	−182	−119	−139	−60	−93
PpcB *Gs* 25	−137	−143	−185	−192	−140	−145	−84	−103
PpcD *Gs* 30	−132	−148	−181	−191	−133	−152	−78	−94
PpcE *Gs* 30	−134	−139	−191	−194	−133	−138	−82	−85

**Figure 8:**
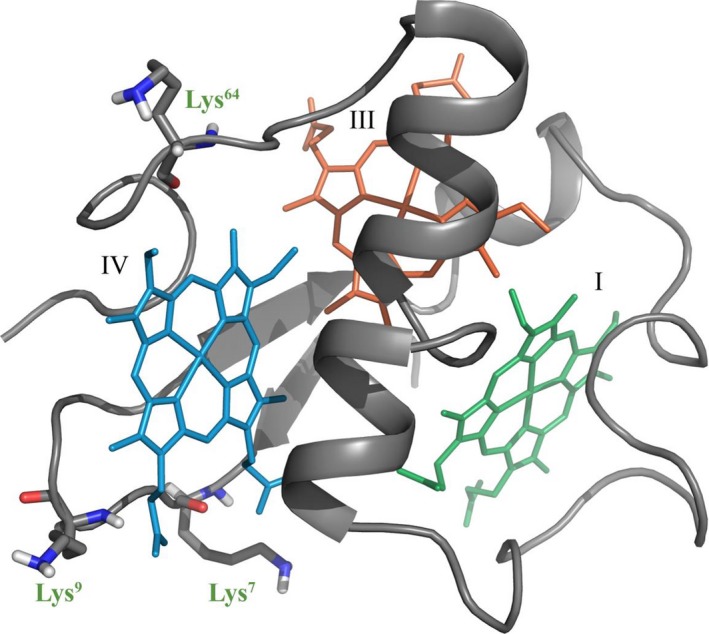
Positively charged residues located in the vicinity of heme IV in the triheme cytochromes from *Geobacter sulfurreducens* but absent in PpcF from *Geobacter metallireducens*. The solution structure of PpcA from *G. sulfurreducens* is used as model (PDB code: http://www.rcsb.org/pdb/search/structidSearch.do?structureId=2LDO 35).

As discussed in the previous point, the order and the profile of oxidation of the heme groups in cytochrome PpcF are quite distinct from those observed for *G. sulfurreducens* cytochromes (Fig. 6). The high oxidation fraction values of heme IV in the first redox step, clearly indicate that the reduced state of this heme is more destabilized compared to the other two hemes. The absence of Lys^9^ and Lys^64^, which are both conserved in *G. sulfurreducens* cytochromes and located in the vicinity of heme IV (Fig. 8), is most likely the reason for the high oxidation fraction values observed for this heme. In fact, the replacement of positively charged lysine residues by noncharged ones in the vicinity of PpcF heme IV is expected to stabilize its oxidized form and thus facilitate its oxidation.

## Conclusions

Nitrate respiration is one of the distinctive biological features of the bacterium *G. metallireducens* compared to the deeply extensively studied *G. sulfurreducens* 37. This work reports the first biochemical characterization of the cytochrome PpcF from *G. metallireducens*, a key protein in this process. The use of complementary spectroscopic techniques, including CD, UV‐visible, and NMR, showed that the cytochrome has three *c*‐type hemes with His‐His axial coordination, being diamagnetic (*S* = 0) and paramagnetic (*S* = 1/2) in the reduced and oxidized states, respectively. The comparison of the heme substituent NMR chemical shifts with those obtained for the homologous cytochromes in *G. sulfurreducens* indicates that the heme core is structurally conserved. However, the functional properties of cytochrome PpcF were found to be significantly different from those observed for its homologues in *G. sulfurreducens*, including the order of oxidation of the heme groups and the working functional redox windows.

The order of oxidation of the hemes observed for PpcF (IV‐I‐III) is unique compared to *G. sulfurreducens* cytochromes. This indicates that the reduced state of heme IV in PpcF is more destabilized, a feature that was explained by the replacement of conserved positively charged residues by noncharged ones, in the vicinity of this heme. In addition, the working redox functional range of cytochrome PpcF is centered at approximately −60 mV, which is significantly less negative compared to the ones observed for *G. sulfurreducens* cytochromes at approximately −130 mV. This constitutes a remarkable example on how similar proteins are differently tuned by their polypeptide chains and optimized to function in specific metabolic pathways. In summary, the redox features displayed by PpcF should pave the way to extend *Geobacter*‐based biotechnological applications to less negative working functional redox windows.

## Author contributions

MRF and JMD carried out the experiments, analyzed the results, and wrote the manuscript; CAS conceived the original idea and supervised the project.

## Conflict of interest

The authors declare no conflict of interest.

## Supporting information


**Fig. S1.** 2D ^1^H,^13^C HMQC NMR spectra of cytochrome PpcF (pH 7.0 and 15 °C). The heme methyl signals are labeled and their chemical shifts are listed in Table S2.
**Table S1.** ^1^H chemical shifts (ppm) of cytochrome PpcF heme substituents in the reduced state at pH 7.0 and 8.0 (15 °C). The values obtained at pH 8.0 are indicated in parenthesis.
**Table S2.** ^1^H and ^13^C chemical shifts (ppm) of the heme methyl groups from cytochrome PpcF in the oxidized state (pH 7.0 and 15 °C).
**Table S3.** Redox‐dependence of the heme methyl chemical shifts, heme oxidation fractions (*x*_*i*_,) and order of oxidation of the hemes in cytochromes PpcA, PpcB, PpcD and PpcE from *G. sulfurreducens* (pH 7.0 and 15 °C). The heme chemical shifts were previously obtained by Morgado and co‐workers [29]. The heme fractions of oxidation, *x*_*i*_, in each oxidation stage were calculated as described in Table 1, accordingly to the equation *x*_*i*_ = (*δ*_*i*_‐*δ*_*0*_)/(*δ*_*3*_‐*δ*_*0*_), where *δ*_*i*_, *δ*_*0*_, and *δ*_*3*_ are the chemical shift of the heme methyls in stages *i*,* 0*, and *3*, respectively. The four oxidation stages are connected by three one‐electron transfer steps that convert the fully reduced (stage *0*) into the fully oxidized (stage *3*) state (for details see main text and Fig. 4).
