# Supplementary material for: The triheme cytochrome PpcF from Geobacter metallireducens exhibits distinct redox properties
Source: FEBS Open Bio. 2018 Nov 8;8(12):1897–910. doi: 10.1002/2211-5463.12505 (PMC6275270; doi:10.1002/2211-5463.12505)
Supplement: Supplementary file 1 — Fig. S1. 2D 1H,13C HMQC NMR spectra of cytochrome PpcF (pH 7.0 and 15 °C). The heme methyl signals are labeled and their chemical shifts are listed in Table S2. Table S1. 1H chemical shifts (ppm) of cytochrome PpcF heme substituents in the reduced state at pH 7.0 and 8.0 (15 °C). The values obtained at pH 8.0 are indicated in parenthesis. Table S2. 1H and 13C chemical shifts (ppm) of the heme methyl groups from cytochrome PpcF in the oxidized state (pH 7.0 and 15 °C). Table S3. Redox‐dependence of the heme methyl chemical shifts, heme oxidation fractions (x i,) and order of oxidation of the hemes in cytochromes PpcA, PpcB, PpcD and PpcE from G. sulfurreducens (pH 7.0 and 15 °C). The heme chemical shifts were previously obtained by Morgado and co‐workers [29]. The heme fractions of oxidation, x i, in each oxidation stage were calculated as described in Table 1, accordingly to the equation x i = (δ i‐δ 0)/(δ 3‐δ 0), where δ i, δ 0, and δ 3 are the chemical shift of the heme methyls in stages i, 0, and 3, respectively. The four oxidation stages are connected by three one‐electron transfer steps that convert the fully reduced (stage 0) into the fully oxidized (stage 3) state (for details see main text and Fig. 4). [file FEB4-8-1897-s001.docx]

**The triheme cytochrome PpcF from *Geobacter metallireducens* exhibits distinct redox properties**

Marisa R. Ferreira, Joana M. Dantas, Carlos A. Salgueiro^*^

UCIBIO-Requimte, Departamento de Química, Faculdade de Ciências e Tecnologia, Universidade NOVA de Lisboa, Campus Caparica, 2829-516 Caparica, Portugal

* Corresponding author: csalgueiro@fct.unl.pt

**ELECTRONIC SUPPLEMENTARY MATERIAL**


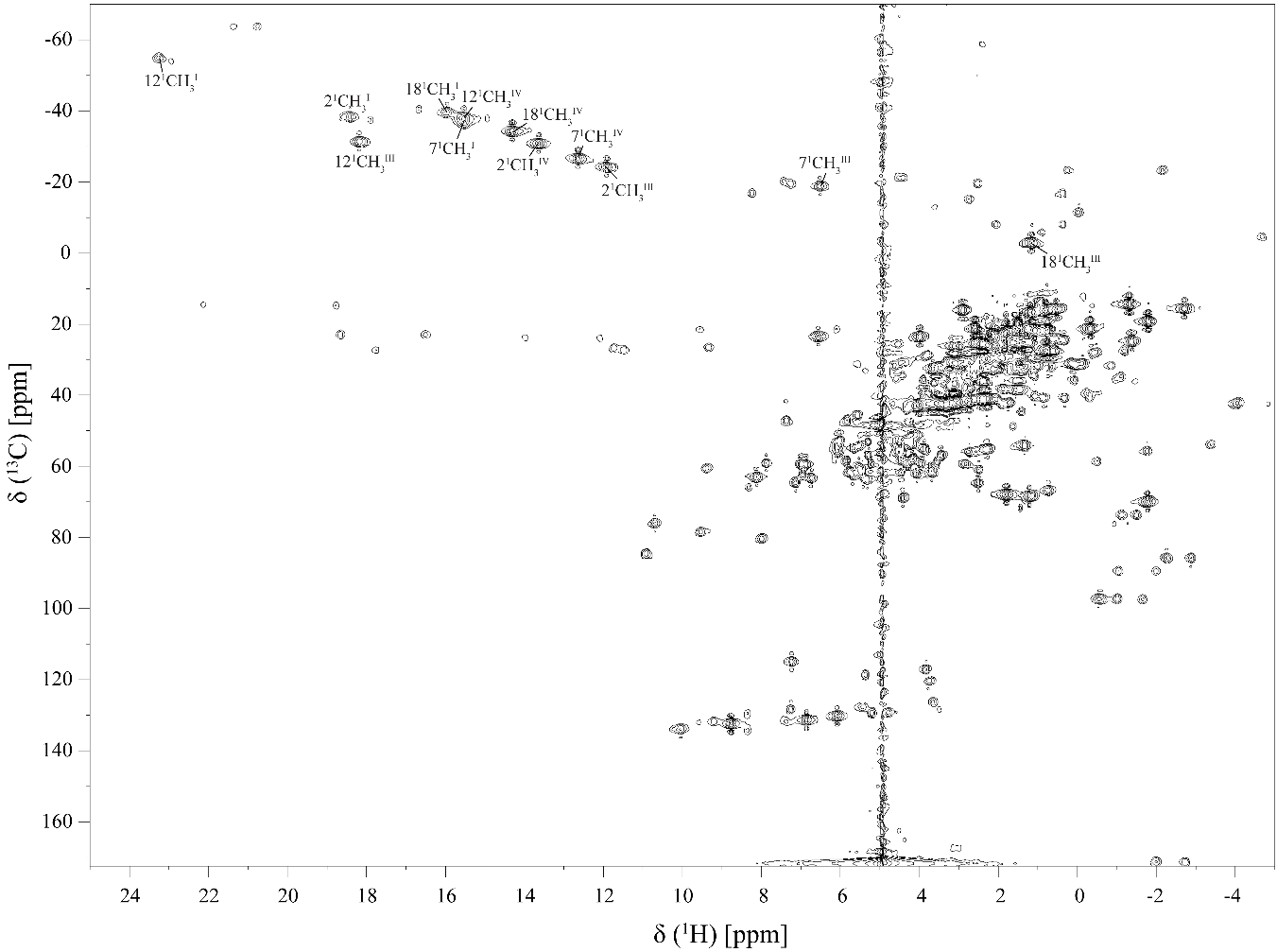


**Figure S1 - 2D ^1^H,^13^C HMQC NMR spectra of cytochrome PpcF (pH 7.0 and 15 °C**). The heme methyl signals are labeled and their chemical shifts are listed in Table S2.

**Table S1. ^1^H chemical shifts (ppm) of cytochrome PpcF heme substituents in the reduced state at pH 7.0 and 8.0 (15 °C).** The values obtained at pH 8.0 are indicated in parenthesis.

| **Heme**  **substituent** | **Chemical shifts (ppm)** | | |
| --- | --- | --- | --- |
|  | **Heme I** | **Heme III** | **Heme IV** |
| 5H | 9.42 (9.41) | 9.77 (9.73) | 9.06 (9.01) |
| 10H | 9.41 (9.39) | 9.78 (9.75) | 9.33 (9.29) |
| 15H | 9.42 (9.30) | 9.51 (9.54) | 9.58 (9.53) |
| 20H | 9.64 (9.60) | 10.26 (10.23) | 9.57 (9.53) |
| 2^1^CH_3_ | 3.42 (3.39) | 4.42 (4.39) | 3.73 (3.70) |
| 7^1^CH_3_ | 3.56 (3.55) | 3.96 (3.92) | 3.06 (3.02) |
| 12^1^CH_3_ | 3.07 (3.09) | 3.51 (3.48) | 3.80 (3.75) |
| 18^1^CH_3_ | 3.61 (3.58) | 4.00 (3.98) | 3.45 (3.42) |
| 3^1^H | 5.85 (5.81) | 6.68 (6.63) | 6.01 (5.96) |
| 8^1^H | 6.52 (6.48) | 6.47 (6.43) | 6.24 (6.21) |
| 3^2^CH_3_ | 2.18 (2.16) | 2.52 (2.48) | 2.04 (2.01) |
| 8^2^CH_3_ | 1.94 (1.92) | 3.00 (2.97) | 1.49 (1.46) |

**Table S2 - ^1^H and ^13^C chemical shifts (ppm) of the heme methyl groups from cytochrome PpcF in the oxidized state (pH 7.0 and 15 °C).**

| **Heme**  **Substituent** | **Chemical shifts (ppm)** | | | | | |
| --- | --- | --- | --- | --- | --- | --- |
|  | **Heme I** | | **Heme III** | | **Heme IV** | |
|  | **^13^C** | **^1^H** | **^13^C** | **^1^H** | **^13^C** | **^1^H** |
| 2^1^CH_3_ | -38.39 | 18.41 | -24.14 | 11.91 | -30.86 | 13.63 |
| 7^1^CH_3_ | -38.06 | 15.54 | -18.88 | 6.50 | -26.69 | 12.63 |
| 12^1^CH_3_ | -55.05 | 23.26 | -31.42 | 18.18 | -37.78 | 15.52 |
| 18^1^CH_3_ | -39.72 | 15.95 | -2.96 | 1.14 | -34.34 | 14.30 |

**Table S3 – Redox-dependence of the heme methyl chemical shifts, heme oxidation fractions (*x_i_*,) and order of oxidation of the hemes in cytochromes PpcA, PpcB, PpcD and PpcE from *G. sulfurreducens* (pH 7.0 and 15 °C).** The heme chemical shifts were previously obtained by Morgado and co-workers [[29](#_ENREF_29)]. The heme fractions of oxidation, *x_i_*, in each oxidation stage were calculated as described in Table 1, accordingly to the equation *x_i_* = (*δ_i_*-*δ_0_*)/(*δ_3_*-*δ_0_*), where *δ_i_*, *δ_0_*, and *δ_3_* are the chemical shift of the heme methyls in stages *i*, *0*, and *3*, respectively. The four oxidation stages are connected three one-electron transfer steps that convert the fully reduced (stage *0*) into the fully oxidized (stage *3*) state (for details see main text and Fig. 4).

|  | Oxidation stage |  | Chemical shifts (ppm) | | | | Oxidation fraction  (*x_i_*) | | | |  | | Order of oxidation |
| --- | --- | --- | --- | --- | --- | --- | --- | --- | --- | --- | --- | --- | --- |
|  |  |  | I | III | IV |  | I | III | IV |  | |  | |
| **PpcA** | *0* |  | 2.55 | 4.14 | 3.95 |  | 0 | 0 | 0 |  | | **I- IV-III** | |
|  | *1* |  | 12.64 | 8.07 | 6.52 |  | 0.53 | 0.28 | 0.17 |  | |  | |
|  | *2* |  | 19.71 | 10.92 | 12.79 |  | 0.90 | 0.48 | 0.59 |  | |  | |
|  | *3* |  | 21.65 | 18.35 | 19.03 |  | 1 | 1 | 1 |  | |  | |
| **PpcB** | *0* |  | 2.73 | 4.14 | 3.61 |  | 0 | 0 | 0 |  | | **III-I-IV** | |
|  | *1* |  | 7.17 | 10.32 | 6.36 |  | 0.29 | 0.53 | 0.17 |  | |  | |
|  | *2* |  | 15.11 | 12.40 | 11.16 |  | 0.80 | 0.71 | 0.47 |  | |  | |
|  | *3* |  | 18.15 | 15.85 | 19.63 |  | 1 | 1 | 1 |  | |  | |
| **PpcD** | *0* |  | 3.23 | 3.92 | 3.38 |  | 0 | 0 | 0 |  | | **I-IV-III** | |
|  | *1* |  | 13.09 | 6.52 | 8.28 |  | 0.44 | 0.21 | 0.36 |  | |  | |
|  | *2* |  | 21.00 | 7.35 | 16.26 |  | 0.79 | 0.28 | 0.94 |  | |  | |
|  | *3* |  | 25.76 | 16.07 | 17.08 |  | 1 | 1 | 1 |  | |  | |
| **PpcE** | *0* |  | 3.30 | 3.20 | 3.63 |  | 0 | 0 | 0 |  | | **III-I-IV** | |
|  | *1* |  | 10.43 | 12.23 | 4.38 |  | 0.42 | 0.53 | 0.05 |  | |  | |
|  | *2* |  | 18.48 | 17.08 | 7.99 |  | 0.90 | 0.82 | 0.29 |  | |  | |
|  | *3* |  | 20.19 | 20.22 | 18.50 |  | 1 | 1 | 1 |  | |  | |
